# Effect of the COVID-19 pandemic on medical student career perceptions: a national survey study

**DOI:** 10.1080/10872981.2020.1798088

**Published:** 2020-07-24

**Authors:** Yasmeen M. Byrnes, Alyssa M. Civantos, Beatrice C. Go, Tara L. McWilliams, Karthik Rajasekaran

**Affiliations:** a Perelman School of Medicine, University of Pennsylvania, Philadelphia, PA, USA; b Biostatistics Analysis Center, University of Pennsylvania, Philadelphia, PA, USA; c Department of Otorhinolaryngology - Head & Neck Surgery, University of Pennsylvania, Philadelphia, PA, USA

**Keywords:** ‘Medical student’, ‘COVID-19’, ‘specialty choice’, ‘virtual education’, ‘career development’

## Abstract

**Background & Objective:**

The COVID-19 pandemic and resulting cancellation of medical student clinical rotations pose unique challenges to students’ educations, the impact of which has not yet been explored.

**Design:**

This cross-sectional survey study collected responses from 13 April 2020 until 30 April 2020. Students at US allopathic medical schools completed the survey online.

**Results:**

1,668 responses were analyzed. A total of 337 (20.2%) respondents thought the pandemic would affect their choice of specialty, with differences across class years: 15.2% (53) of first-years (MS1s), 26.4% (92) of second-years (MS2s), 23.7% (162) of third-years (MS3s), and 9.7% (22) of fourth-years (MS4s) (p < 0.0001). Among all classes, the most common reason chosen was inability to explore specialties of interest (244, 72.4%), and the second was inability to bolster their residency application (162, 48.1%). Out of the MS3s who chose the latter, the majority were concerned about recommendation letters (68, 81.0%) and away rotations (62, 73.8%). As high as 17.4% (119) of MS3s said they were more likely to take an extra year during medical school as a result of the pandemic. Region of the US, number of local COVID cases, and number of local COVID deaths had no effect on whether respondents thought the pandemic would affect their specialty choice.

**Conclusions:**

Our study found that about one-fifth of surveyed medical students currently believe that the COVID-19 pandemic will affect their choice of specialty, with many of these citing concerns that they cannot explore specialties or obtain recommendation letters. With prolonged suspension of clinical rotations, targeted efforts by medical schools to address these concerns through enhanced virtual curriculum development and advising strategies will become increasingly important. Further study is needed to explore whether these cross-sectional student perspectives will manifest as changes in upcoming National Residency Matching Program data.

## Introduction

The global pandemic caused by SARS-CoV-2 has caused unprecedented changes to almost every aspect of society, with social distancing orders taking effect across the USA (US) beginning in March 2020 [1,2].⁠ Medical student education has been uniquely impacted by these changes. For the vast majority of medical students across the country, all clinical learning has been indefinitely suspended in accordance with the American Association of Medical Colleges (AAMC) recommendations on 17 March 2020 [3],⁠ including Visiting Student Learning Opportunities (away rotations)[4].⁠ Many medical schools have now developed virtual curricula to continue medical education until students are able to return to hospitals and clinics [5–9].^⁠^ However, lack of in-person clinical exposure may pose a challenge to medical students, particularly those attempting to choose a specialty or apply to residency programs. Published commentaries by medical students have indicated a simultaneous sense of duty to do their part by staying home, as well as concern about how their education will proceed [10,11].⁠

Several previous disruptions to medical school clinical curriculum and their effects have been described in the literature. During part of the 2002–2004 Severe Acute Respiratory Syndrome (SARS) pandemic, medical students in affected countries were barred from direct in-person patient care [12–14].⁠ At these institutions, alternative virtual coursework was adopted, such as e-learning modules, video vignettes, and virtual reality simulators [12].⁠ Similar measures were adopted in certain regions during the Middle Eastern Respiratory Syndrome (MERS) pandemic [15].⁠ When Hurricane Katrina struck New Orleans, Tulane University Medical School relocated medical student education, including clinical rotations, to four medical schools in Houston, Texas [16].⁠ While to our knowledge there are no studies exploring SARS or MERS impact on medical student specialty choice, one post-Katrina New Orleans study found that medical students were significantly more likely to specialize in Emergency Medicine (EM) and less likely to specialize in Psychiatry in the 5 years after the hurricane than in the 5 years before [17].⁠

Changes in medical education in response to these disasters, while locally disruptive, were not as widespread as the changes brought on by the COVID-19 pandemic. The current changes to US medical education are unprecedented. Although there are some existing anecdotal commentaries describing effects of the COVID-19 pandemic on medical education [5,18–21],^⁠^ studies describing virtual curriculum development [5–8], and reports of medical student mobilization in response to the pandemic [22–26],⁠ to our knowledge no data yet exists describing the current impact on medical students’ academic lives. It is not yet known how long the suspension of clinical rotations will continue[27].⁠ As such, early identification of concerns and potential issues may help guide career advising, virtual curriculum development, and general support for medical students during this unique time. This study aimed to provide a cross-sectional view into the impact of the COVID-19 crisis on US medical students’ perception of their education and career development.

## Methods

### Study design

This cross-sectional online survey study collected response data from medical students across the USA from 13 April 2020 through 30 April 2020. The survey was designed by three investigators (YB, AC, and KR) and contained 9 multiple choice items with up to 6 possible follow-up multiple choice items depending on the participant’s answers (see Appendix 1). It contained no identifying information. Respondent demographics included US state in which the respondent attended medical school and class year. The survey gave students the option of selecting up to two specialties of interest. A variable for certainty of specialty was created using these responses. Those who selected only one specialty were considered certain. Those who selected either two specialties or chose the option ‘undecided’ were considered uncertain. The survey also asked students which electives they had completed if any. Using their response to this in conjunction with choice of specialties, a variable was created to indicate whether students had taken an elective in their specialty of interest. Those whose specialty was listed as ‘undecided’, those who had not taken any electives, and those who had taken electives that did not match their specialty of interest were considered as not having taken an elective in their specialty of interest.

Prior to national distribution, feedback on survey design and content was solicited and incorporated from 7 home-institution medical students with a variety of specialty interests via open-ended verbal discussions (one 3-person group discussion and four 1-on-1 discussions). Responses to the survey were collected and managed using REDCap electronic data capture tools [28,29].⁠ The survey was self-administered and accessed by participants via an electronic link. Participation was voluntary, and participants were allowed to terminate the survey at any time. This project was reviewed and determined to qualify as quality improvement by the University of Pennsylvania Institutional Review Board.

### Survey distribution

The survey was distributed nationally using a multi-pronged approach. A list of all 150 American Association of Medical Colleges (AAMC) medical schools in the continental USA, Hawaii, and Alaska was generated [30].⁠ Canadian, Caribbean, and Puerto Rican AAMC medical schools were excluded from this list. For each school on the list, Deans or Directors of Student Affairs were contacted via email. They were given a description of the study and asked to distribute the survey link to their institution’s entire medical student body. We did not have a method by which to confirm which Deans or Directors actually distributed it. In addition to this approach, based on investigators’ knowledge of existing communication structures among students, additional medical student-led distribution was also achieved at many schools through email listservs and social media groups.

### Statistical analysis

All analyses were conducted using SAS version 9.4 (SAS Institute, Cary, NC). Cross-tabulated frequencies and percentages were calculated, and all associations were quantified using chi-squared and Fisher’s exact tests, as applicable. Fisher’s exact tests were used in cases where a combination of two variables resulted in any cell counts of five or less. A p-value equal to or less than 0.05 was considered to indicate a statistically significant relationship between two variables.

State of medical school location was categorized as one of four USA regions (Northeast, South, Midwest, and West) using guidelines from the US Census Bureau [31].⁠ Publicly available COVID-19 data were used as proxy measures for pandemic severity in each US state. COVID-19 confirmed cumulative positive case counts and cumulative death counts by state as of 21 April 2020 at 4pm EST were acquired from The COVID Tracking Project [32]. This date was chosen because it was halfway through our 18-day study period. The number of positive cases in each state was originally a continuous variable with median 19,881. Using this, number of cases was dichotomized into roughly two equal groups, at least 19,000 cases and under 19,000 cases. Similarly, the median number of deaths was 584 and was dichotomized into at least 600 deaths and under 600 deaths.

## Results

### Descriptive statistics

1668 responses were analyzed, from 20 US states [Table 1]. The majority of responses were from the Northeast (623, 37.4%), South (454, 27.2%), and Midwest (530, 31.8%), with a minority (61, 3.7%) from the West. A plurality of respondents were MS3s (684, 41.0%), with 349 (20.9%) MS1s, 349 (20.9%) MS2s, and 226 (13.6%) MS4s. A small minority of respondents (61, 3.7%) were MD-PhDs within the research part of their program, students taking a year out during medical school for research, dual degree, or other reasons, or ‘other’. For simplicity of analysis, these respondents were collapsed into one category titled ‘Other’.

**Table 1.** Number of responses by state^a.^

State	Responses
Missouri	242
New York	217
Pennsylvania	172
Ohio	170
Maryland	156
Georgia	98
Texas	98
Massachusetts	79
New Hampshire	76
Louisiana	48
California	46
Vermont	45
Illinois	43
Wisconsin	41
Connecticut	34
Virginia	33
South Carolina	21
North Dakota	20
Utah	15
Michigan	14
**Total**	**1668**

^a^Responses from states with <10 total responses were completely excluded from all analyses so as to ensure adequate representation from included states.

Respondents’ level of clinical exposure (as measured by whether they had completed, not started, or partially completed core rotations) differed across class years (p < 0.0001) [Table 2]. A majority of MS2s had not yet begun clerkship year (222, 63.6%), while the majority of MS3s had begun the year but not completed it (439, 64.2%). In total there were 606 (36.3%) students who were part-way through clerkship year when the pandemic started. Out of these, roughly half of the MS2s had not completed either surgery or internal medicine (69, 55.2%), while about the same proportion of MS3s had completed both (255, 58.1%).

**Table 2.** Core rotation completion status by class year^a.^

	MS1^b^ N = 348	MS2 N = 349	MS3 N = 684	MS4 N = 226	Other N = 61	Total N = 1668	P-value
**Core Rotations** All completed Some completed Not started	0 0 348 (100)	2 (0.6) 125 (35.8) 222 (63.6)	224 (32.8) 439 (64.2) 21 (3.1)	199 (88.1) 27 (12.0) 0	27 (44.3) 15 (24.6) 19 (31.2)	452 (27.1) 606 (36.3) 610 (36.6)	<0.0001
**Core Rotations Completed^c^** Internal med. no surgery Surgery, no internal med. Neither Both	– – – –	31 (24.8) 25 (20.0) 69 (55.2) 0	79 (18.0) 74 (16.9) 31 (7.1) 255 (58.1)	0 1 (3.7) 0 26 (96.3)	3 (20.0) 2 (13.3) 3 (20.0) 7 (46.7)	113 (18.7) 102 (16.8) 103 (17.0) 294 (47.5)	<0.0001

^a^Values are presented as N (%). Percentages are column percentages.

^b^Abbreviations: MS1 = first year student, MS2 = second year student, MS3 = third year student, MS4 = fourth year student

^c^Only includes those who have completed some, but not all, of their core rotations. N = 606.

### Pandemic effect on specialty choice

The percentage of respondents who were certain about their specialty choice differed significantly across classes; 64.5% (441) of MS3s were certain, as opposed to 16.1% (56) of MS2s and 14.4% (50) of MS1s (p < 0.0001). When asked to indicate up to two specialties they were ‘seriously considering’, MS3s were considering a broad range of options, with Internal Medicine (IM), Pediatrics, and Emergency Medicine (EM) being the most commonly chosen [Table 3].

**Table 3.** Specialties 3^rd^ year medical students were seriously considering^a.^

	Count	Percentage
**Internal Medicine**	169	24.7
**Pediatrics**	102	14.9
**Emergency Medicine**	98	14.3
**Obstetrics & Gynecology**	83	12.1
**General Surgery**	64	9.4
**Anesthesiology**	53	7.7
**Orthopaedic Surgery**	49	7.2
**Psychiatry**	44	6.4
**Family Medicine**	40	5.8
**Otolaryngology**	31	4.5
**Neurology**	28	4.1
**Medicine/Pediatrics**	27	3.9
**Ophthalmology**	22	3.2
**Radiology (Diagnostic)**	20	2.9
**Urology**	18	2.6
**Dermatology**	16	2.3
**Plastic Surgery**	15	2.2
**Child Neurology**	11	1.6
**Pathology**	8	1.2
**Neurosurgery**	7	1.0
**Physical Medicine & Rehabilitation**	7	1.0
**Radiation Oncology**	5	0.7
**Interventional Radiology**	3	0.4
**Thoracic Surgery**	3	0.4
**Undecided**	2	0.3
**Vascular Surgery**	1	0.1

^a^Respondents were allowed to indicate either one or two specialties for this survey item.

As high as 20.2% (337) of medical students surveyed thought that the pandemic would affect their choice of specialty. There was a significant difference across class years, with 15.2% (53) MS1s, 26.4% (92) MS2s, 23.7% (162) MS3s, and 9.7% (22) of MS4s saying their specialty choice would be affected (p < 0.0001). Across all class years, the most common reason chosen was ‘I may not have the opportunity to explore my specialty or specialties of interest’ (244, 72.4%). The majority of MS3s had not done an elective in their specialty of choice (601, 87.8%). However, on further analysis of MS3s, there was no significant relationship between completion of an elective of interest and certainty in specialty choice. As high as 12.7% (56) of MS3s who were certain about specialty choice had done an elective in their specialty of interest as compared to 11.1% (27) of MS3s who were uncertain about specialty choice (p = 0.54).

Out of those who thought the pandemic would affect their choice of specialty, the second most common reason given was, across all classes, ‘I no longer have the ability to bolster my application’ (162, 48.1%). When asked what they meant, MS3s who chose this reason were most commonly concerned about ‘letters of recommendation’ (68, 81.0%) and ‘away rotations’ (62, 73.8%), whereas MS2s most often chose ‘taking board exams’ (27, 71.1%), and MS1s most often chose ‘research’ (24, 100.0%). When MS3s were asked whether the COVID-19 pandemic has made them more likely to take an extra year in medical school before applying to residency, 119 (17.4%) said yes. Out of these, the most common reason given was ‘it would make me more likely to match to my satisfaction’ (79, 66.4%). Three hundred and twenty-seven (47.8%) of MS3s and 55 (24.3%) of MS4s were concerned about fulfilling graduation requirements in time.

Respondents who thought the pandemic would affect their choice of specialty were asked, as a follow-up, what specialties they were ‘more likely’ and ‘less likely’ to apply into, respectively. No clearly favored or disfavored specialties emerged. IM was the most common answer for ‘more likely’, while EM was the most commonly chosen as ‘less likely’. However, EM was also the second most common choice put for ‘more likely’, and IM was the second most common choice put for ‘less likely’.

An analysis using proxy measures for COVID-19 severity level near where respondents attended medical school was done. Region of the US, number of statewide COVID cases, number of statewide COVID deaths all had no correlation with the proportion of respondents who thought the pandemic would affect their specialty choice (p = 0.17, 0.25, and 0.21, respectively).

### Activities while away from clinical rotations

When asked how they were spending their time away from the clinics [Table 4], ‘research’ and ‘virtual classes through medical school’ were the only academic-related options selected by greater than half of respondents. Five hundred and nine (30.5%) and 333 (20.0%) indicated involvement in service work or community engagement/organizing, respectively. In the non-academic category, ‘hobbies’, ‘exercise’, and ‘self care/relaxing’ were each selected by greater than half of respondents. Our survey also included an optional comment box. Relevant comments will be summarized in the following section as qualitative information to help lend nuance to our discussion of the results.

**Table 4.** Activities medical students are engaged in during the pandemic by class year^a.^

	MS1 N = 348	MS2 N = 349	MS3 N = 684	MS4 N = 226	Other N = 61	Total N = 1668
**Online classes through med school**	338 (97.1)	226 (64.8)	574 (83.9)	125 (55.3)	18 (29.5)	1281 (76.8)
**Self care/relaxing**	266 (76.4)	229 (65.6)	506 (74.0)	183 (81.0)	41 (67.2)	1225 (73.4)
**Exercise**	256 (73.6)	247 (70.8)	513 (75.0)	172 (76.1)	33 (54.1)	1221 (73.2)
**Hobbies**	227 (65.2)	198 (56.7)	459 (67.1)	175 (77.4)	38 (62.3)	1097 (65.8)
**Research**	139 (39.9)	141 (40.4)	420 (61.4)	107 (47.4)	52 (85.3)	859 (51.5)
**Family responsibilities**	193 (55.5)	161 (46.1)	307 (44.9)	115 (50.9)	19 (31.2)	795 (47.7)
**Service work**	81 (23.3)	84 (24.1)	261 (38.2)	64 (28.3)	19 (31.2)	509 (30.5)
**Seeking academic advising**	36 (10.3)	54 (15.5)	248 (36.3)	41 (18.1)	11 (18.0)	390 (23.4)
**Community engagement or organizing**	70 (20.1)	51 (14.6)	166 (24.3)	31 (13.7)	15 (24.6)	333 (20.0)
**Other online classes**	45 (12.9)	74 (21.2)	140 (20.5)	43 (19.0)	17 (27.9)	319 (19.1)
**Telemedicine work**	23 (6.6)	34 (9.7)	138 (20.2)	23 (10.2)	10 (16.4)	228 (13.7)
**Networking**	31 (8.9)	21 (6.0)	72 (10.5)	18 (8.0)	5 (8.2)	147 (8.8)
**Preparing for board exams**	0	72 (20.6)	26 (3.8)	9 (4.0)	0	107 (6.4)

^a^Respondents were allowed to select more than one answer. Values are presented as N (%). Percentages are column percentages.

## Discussion

This study serves as an early snapshot into medical students’ perspectives on their education and careers at a time when the COVID-19 pandemic is in full force and clinical rotations remain cancelled. Despite substantial curriculum upheaval, only about one-fifth of our respondents indicated that their specialty choice would be affected. Most of these students cited concerns about not having time to explore their specialties of interest.

Medical student specialty choice has been written about prior to the COVID-19 pandemic, and studies have suggested many factors can play a role, such as exposure to role models and core rotation experiences[33].⁠ Jones et al. found that, as one might expect, the positive predictive value of a medical student’s top specialty choice increases from end of first year (ranging from 17% to 60% depending on specialty) through end of third year (ranging from 79% to 95%), suggesting that more exposure to fields of potential interest throughout medical school may result in increased accuracy of self-predictions. They also found that students’ self-reported level of certainty was not correlated with the positive predictive value of their specialty choice[34].⁠ On the other hand, Manuel found that there was a significant relationship between first-semester medical students’ predictions of whether they would apply into a technique-oriented or person-oriented specialty and their eventual choice[35].⁠ At baseline (pre-pandemic), though they might have an idea of the broad category of career they prefer, MS1s, MS2s, and even MS3s can be inaccurate at predicting their future specialty choice regardless of whether they feel certain.

Our results found that 9.7% (22) of MS4s thought the pandemic would affect their specialty choice, an odd finding considering that by April, when our survey was distributed, MS4s have generally already matched or not matched into a single specialty of their choice. There are a couple possible explanations for this finding. Some MS4s may have chosen not to apply to residency. Also, students taking a year out during medical school between 3^rd^ and 4^th^ year may have identified themselves as MS4s rather than year out students.

In our survey, students who indicated that they did not think their specialty choice would be affected were not given follow-up questions asking about their specific concerns. However, many of them independently brought up some of the same concepts in the optional comment box at the end of the survey. Though these respondents did not think their choice would be affected, many were still concerned about their ability to be as competitive in their chosen specialty as they otherwise would have been, and submitted comments about away rotations, letters of recommendation, completing board exams in time, and inadequate clinical preparedness due to virtual coursework being a poor substitute for rotations. Literature on virtual learning in medical students prior to the COVID-19 pandemic have found that though students feel it augments their education, they do not view it as an adequate replacement for in-person learning [36,37].⁠

By now, many medical schools have created virtual coursework so that students can continue learning from home [5–9,20].⁠ Literature on the precise structure and content of coursework being created at different institutions is limited thus far, but given that course development is not centralized on a national level, one can only imagine it varies greatly from school to school in terms of fidelity to a true clinical rotation, level of interactivity, presence of virtual patient contact, or quality of contact with residents and faculty. As such, these curricula likely also vary in terms of how well they address the concerns described in our results, particularly obtaining letters of recommendation, gaining clinical preparedness, and accurately evaluating specialty fit. It is still unclear when medical students across the US will be able to return to clinical rotations[27].⁠ The longer that rotations remain canceled, the more crucial it will become to carefully examine which types of virtual curricula, if any, are effective at addressing medical students’ career-related concerns.

Our results show that students are currently filling their time with a variety of personal and professional endeavors, most notably, research and the previously mentioned virtual coursework. Although medical student volunteering and community-based efforts to combat COVID-19 have been frequently featured in the news and described in the literature [4,22,24,38,39],⁠ our data indicate that only about half of students are involved with these endeavors, which may be less than the common perception.

The severity of the COVID-19 pandemic in the US state in which medical students attended school did not have an effect on their perception of the pandemic’s effect on their careers. This is a logical finding; local climate may certainly affect other aspects of students’ lives such as magnitude of student-led volunteer efforts [24,39],⁠ and concern for safety of self and loved ones. However, if medical students have concerns about their education and career choices, these are most likely to stem from the sudden and drastic disruption to their curriculum [11,18,20].⁠ Furthermore, our analysis of measures of pandemic severity was at the state level. It is possible that a city-level analysis would produce different results, as it is becoming increasingly clear that COVID pandemic severity often varies across different cities within states [40,41].⁠

Strengths of this study include a large sample size, a wide geographical distribution, and timely development and distribution. However, several limitations must be discussed as well, particularly the possibility of response bias. Students who are willing to take surveys may be a group with different attitudes toward the pandemic and toward career choices than those who are not willing to take surveys. Since our survey was also distributed via medical student social media groups, it is also possible that students who regularly engage with these groups on social media may have different traits and perceptions as well. In addition, to maximize anonymity, our survey did not prompt respondents to include their medical school name (merely the state in which they attended medical school); thus, we were not able to track which institutions out of those we contacted actually distributed the survey.

Our early cross-sectional study did not identify any specialties that were clearly favored or disfavored as a result of the pandemic. Future study will be necessary to determine whether the thought processes and concerns revealed in our results manifest as real changes in National Residency Matching Program (NRMP/the Match) data, or whether they are transient issues that resolve as students are allowed to resume rotations. In the aforementioned study on medical student specialty choice before and after Hurricane Katrina (another public health event that disrupted medical education), there were significant changes in specialty choice before and after the event[17].⁠ However, this change was evident over a 10-year study period. A similar long-term analysis of medical student specialty choices before and after the COVID-19 pandemic would certainly be a worthwhile study in the future.

## Conclusion

The COVID-19 pandemic has disrupted clinical education at medical schools across the US. Our results indicate that though the majority of medical students do not think this disruption will affect their specialty choice, about one in five do. It is impossible to tell at this early stage whether the career-related concerns revealed by our findings will manifest as changes in residency Match data. What we can conclude is that the longer the COVID-19 pandemic prevents medical students from completing clinical rotations, the more important it will become for medical schools to address these concerns in a targeted manner through enhanced virtual coursework and advising support.

## Supplementary Material

Supplemental Material

## Data Availability

The dataset supporting the conclusions of this article is attached as a supplemental Excel file.

## Authors’ information

YB, AC, and BG are medical students, Perelman School of Medicine, University of Pennsylvania, Philadelphia, PA. TM is a statistical analyst, Biostatistics Analysis Center, University of Pennsylvania, Philadelphia, PA. KR is an assistant professor, Department of Otorhinolaryngology - Head & Neck Surgery, University of Pennsylvania, Philadelphia, PA.

## Declarations

**Ethics approval and consent to participate**: This project was reviewed and determined to qualify as quality improvement by the University of Pennsylvania’s Institutional Review Board.

## Appendix 1. Full Copy of Survey

**Question 1: What year in medical school are you currently in?**

(a)MS1

(b)MS2

(c)MS3

(d)MS4

(e)Year Out

(f)PhD part of MD-PhD

(g)Other – please specify ______

**Question 2. Which of the following statements is most correct?**

(a)I have completed all of my core rotations.

(b)I have completed some of my core rotations but not all of them.

(c)I have not started my core rotations.

(d)Other

If and only if they chose (b) for Question 2, they are directed to Question 2a:

**Question 2b: Which core rotations have you completed? (You may select more than one.)**

(a)Surgery

(b)Internal Medicine

(c)OB/Gyn

(d)Pediatrics

(e)Psychiatry

(f)Family Medicine

(g)Neurology

(h)Emergency Medicine

(i)Radiology

(j)Other – please specify ______

**Question 3. What state is your medical school in?**

(a)Alabama

(b)Alaska

(c)Arizona

(d)Arkansas

(e)California

(f)Colorado

(g)Connecticut

(h)Delaware

(i)Florida

(j)Georgia

(k)Hawaii

(l)Idaho

(m)Illinois

(n)Indiana

(o)Iowa

(p)Kansas

(q)Kentucky

(r)Louisiana

(s)Maine

(t)Maryland

(u)Massachusetts

(v)Michigan

(w)Minnesota

(x)Mississippi

(y)Missouri

(z)Montana

(aa)Nebraska

(ab)Nevada

(ac)New Hampshire

(ad)New Jersey

(ae)New Mexico

(af)New York

(ag)North Carolina

(ah)North Dakota

(ai)Ohio

(aj)Oklahoma

(ak)Oregon

(al)Pennsylvania

(am)Rhode Island

(an)South Carolina

(ao)South Dakota

(ap)Tennessee

(aq)Texas

(ar)Utah

(as)Vermont

(at)Virginia

(au)Washington

(av)West Virginia

(aw)Wisconsin

(ax)Wyoming

(ay)DC

(az)Other – please specify ______

**Question 4. What specialty or specialties are you currently seriously considering? (You may choose up to 2 answers.)**

1. Anesthesiology
2. Child Neurology
3. Dermatology
4. Emergency Medicine
5. Family Medicine
6. General Surgery
7. Internal Medicine
8. Interventional Radiology (Integrated)
9. Medical Genetics
10. Medicine/Pediatrics
11. Neurosurgery
12. Neurology
13. Nuclear Medicine
14. Obstetrics & Gynecology
15. Ophthalmology
16. Orthopaedic Surgery
17. Otolaryngology
18. Pathology
19. Pediatrics
20. Physical Medicine & Rehabilitation
21. Plastic Surgery (Integrated)
22. Preventive Medicine
23. Psychiatry
24. Radiology (Diagnostic)
25. Radiation Oncology
26. Thoracic Surgery (Integrated)
27. Urology
28. Vascular Surgery (Integrated)
29. Undecided
30. Other – please specify ________

**Question 5. Have you completed any electives or sub-internships?**

(a)Yes

(b)No

If and only if they chose (a) for Question 5, they are directed to Question 5a:

**Question 5a: Which electives or subs have you completed? (You may choose up to 3 answers.)**

(a)Anesthesiology

(b)Child Neurology

(c)Dermatology

(d)Emergency Medicine

(e)Family Medicine

(f)General Surgery

(g)Internal Medicine

(h)Interventional Radiology (Integrated)

(i)Medical Genetics

(j)Medicine/Pediatrics

(k)Neurosurgery

(l)Neurology

(m)Nuclear Medicine

(n)Obstetrics & Gynecology

(o)Ophthalmology

(p)Orthopaedic Surgery

(q)Otolaryngology

(r)Pathology

(s)Pediatrics

(t)Physical Medicine & Rehabilitation

(u)Plastic Surgery (Integrated)

(v)Preventive Medicine

(w)Psychiatry

(x)Radiology (Diagnostic)

(y)Radiation Oncology

(z)Thoracic Surgery (Integrated)

(aa)Urology

(ab)Vascular Surgery (Integrated)

(ac)Other – please specify ________

**Question 6. Do you think the COVID-19 pandemic will affect your choice of specialty?**

(a)Yes

(b)No

If and only if they chose (a) for Question 6, they are directed to Question 6a:

**Question 6a: If you answered yes to the above question, why? (You may select more than one.)**

(a)I may not have the opportunity to explore my specialty or specialties of interest

(b)I have discovered new interests or priorities

(c)I no longer have the ability to bolster my application

(d)Other – please specify _______

If and only if they chose (c) for Question 6a, they are directed toQuestion 6a(i):

**Question 6a(i): What part(s) of your application are you concerned about? (You may select more than one.)**

(a)Away rotations

(b)Research

(c)Letters of recommendation

(d)Networking

(e)Taking board exams

(f)Other – please specify ______

If and only if they chose (a) for Question 6, they are directed to Question 6b:

**Question 6b: Which specialty are you *more* likely to apply into?**

(a)Anesthesiology

(b)Child Neurology

(c)Dermatology

(d)Emergency Medicine

(e)Family Medicine

(f)Internal Medicine

(g)Interventional Radiology (Integrated)

(h)Medical Genetics

(i)Medicine/Pediatrics

(j)Neurological Surgery

(k)Neurology

(l)Nuclear Medicine

(m)Obstetrics & Gynecology

(n)Ophthalmology

(o)Orthopaedic Surgery

(p)Otolaryngology

(q)Pathology (Anatomic & Clinical)

(r)Pediatrics

(s)Physical Medicine & Rehabilitation

(t)Plastic Surgery (Integrated)

(u)Preventive Medicine

(v)Psychiatry

(w)Radiation Oncology

(x)Radiology (Diagnostic)

(y)Surgery

(z)Thoracic Surgery (Integrated)

(aa)Urology

(ab)Vascular Surgery (Integrated)

(ac)Other – please specify ______

If and only if they chose (a) for Question 6, they are directed to Question 6 c:

**Question 6 c: Which specialty are you *less* likely to apply into?**

(same options as Question 6 c)

**Question 7. Are you concerned about fulfilling your graduation requirements in time?**

(a)Yes

(b)No

**Question 8. Has the COVID-19 pandemic made you more likely to take an extra year in medical school before applying to residency?**

(a)Yes

(b)No

If and only if they chose (a) for Question 8, they are directed to Question 8a:

**Question 8a: Why?**

(a)It would give me more time to explore different specialties

(b)It would make me more likely to match to my satisfaction

(c)I may not be able to meet my graduation requirements otherwise

(d)I want to use the extra year to explore new interests that developedduring the pandemic

(e)Other – please specify _______

**Question 9. What activities are you currently engaged in during this time away from the hospital? (You may select more than one.)**

(a)Telemedicine work

(b)Service work

(c)Community engagement or organizing

(d)Research

(e)Networking

(f)Seeking academic advising

(g)Online/virtual classes through your medical school

(h)Other online/virtual classes

(i)Family responsibilities

(j)Hobbies

(k)Exercise

(l)Self care/relaxing

(m)Preparing for board exam

(n)Other – please specify ______

**Question 10: Any additional thoughts or comments (optional)**

(Blank box for optional write in responses.)

**Table:**

This document was exported from Numbers. Each table was converted to an Excel worksheet. All other objects on each Numbers sheet were placed on separate worksheets. Please be aware that formula calculations may differ in Excel.		
Numbers Sheet Name	Numbers Table Name	Excel Worksheet Name
Sheet 1		
	Table 1	Sheet 1

## References

[1] Trump Extends Social Distancing Guidelines Through End of April - The New York Times. cited 2020 55. Available from: https://www.nytimes.com/2020/03/29/us/politics/trump-coronavirus-guidelines.html

[2] Social Distancing. Quarantine, and Isolation. cited 2020 55. Available from: https://www.cdc.gov/coronavirus/2019-ncov/prevent-getting-sick/social-distancing.html

[3] Important Guidance for Medical Students on Clinical Rotations During the Coronavirus (COVID-19) Outbreak | AAMC. cited 2020 55. Available from: https://www.aamc.org/news-insights/press-releases/important-guidance-medical-students-clinical-rotations-during-coronavirus-covid-19-outbreak

[4] Coronavirus (COVID-19) and The VSLO Program. cited 2020 59. Available from: https://students-residents.aamc.org/attending-medical-school/article/coronavirus-covid-19-and-vslo-program/

[5] Calhoun KE, Yale LA, Whipple ME, et al. The impact of COVID-19 on medical student surgical education: implementing extreme pandemic response measures in a widely distributed surgical clerkship experience. Am J Surg. 2020 4. Published online. DOI:10.1016/j.amjsurg.2020.04.024. PMC7186124 32389331

[6] Mukhopadhyay S, Booth AL, Calkins SM, et al. Leveraging technology for remote learning in the era of COVID-19 and social distancing: tips and resources for pathology educators and trainees. Arch Pathol Lab Med. 2020 5 4. Published online, :arpa.2020-0201-ED. . DOI:10.5858/arpa.2020-0201-ED. 32364793

[7] Evans DJR, Bay BH, Wilson TD, et al. Going virtual to support anatomy education: A STOP GAP in the midst of the Covid-19 pandemic. Anat Sci Educ. 2020 4 10. Published online. DOI:10.1002/ase.1963. 32277598

[8] Srinivasan DK. Medical students’ perceptions and an anatomy teacher’s personal experience using an e-learning platform for tutorials during the Covid-19 crisis. Anat Sci Educ. 2020 5 6. Published online. DOI:10.1002/ase.1970. 32374937

[9] Haroian N, Pirlamarla P, Lev Y et al. . Outpatient clinical student rotation without direct patient contact: leveraging care transitions to benefit students and patients. *AAMC iCollaborative*. cited 2020 59. Available from: https://icollaborative.aamc.org/resource/5072/

[10] Menon A, Klein EJ, Kollars K, et al. Medical students are not essential workers: examining institutional responsibility during the COVID-19 pandemic. Acad Med. 2020 4 28. Published online. DOI:10.1097/ACM.0000000000003478. PMC7202103 32349014

[11] Theoret C, Our Education MX. Our concerns: medical student education impact due to COVID‐19. Med Educ. 2020 4 20. Published online. DOI:10.1111/medu.14181. PMC7264564 32310318

[12] Lim ECH, Oh VM, Koh DR, et al. The challenges of “continuing medical education” in a pandemic era. Ann Acad Med Singapore. 2009;38(8):724–9. 19736579

[13] McGugan S. Medical school on bypass during the SARS outbreak. Clin Invest Med. 2003;26(3):106. 12858941

[14] Patil NG, Ho C, Yan Y. SARS and its effect on medical education in Hong Kong. Med Educ. 2003;37(12):1127–1128. 14984121 10.1046/j.1365-2923.2003.01723.x PMC7168501

[15] Park SW, Jang HW, Choe YH, et al. Avoiding student infection during a Middle East respiratory syndrome (MERS) outbreak: a single medical school experience. Korean J Med Educ. 2016;28(2):209–217. 27240893 10.3946/kjme.2016.30 PMC4951746

[16] Searle NS, Greenberg SB, Andrews LB, et al. Baylor college of medicine’s support of tulane university school of medicine following Hurricane Katrina. Acad Med. 2007;82(8):733–744. 17762246 10.1097/ACM.0b013e3180d0964f

[17] Townsend MH. Snap shots: the effect of Hurricane Katrina on medical student career choice. Acad Psychiatry. 2012 5 1;36(3):258–259. . 22751837 10.1176/appi.ap.11030056

[18] Rose S. Medical student education in the time of COVID-19. JAMA. 2020 3 31. Published online. DOI:10.1001/jama.2020.5227. 32232420

[19] Whelan AJ. The change to pass/fail scoring for step 1 in the context of COVID-19. Acad Med. 2020 4:1. Published online. Doi:10.1097/acm.0000000000003449. 32324640 PMC7192359

[20] No classrooms, no clinics: Medical education during a pandemic | AAMC. cited 2020 59. Available from: https://www.aamc.org/news-insights/no-classrooms-no-clinics-medical-education-during-pandemic

[21] Ferrel MN, Ryan JJ. The impact of COVID-19 on medical education. Cureus. 2020;12:3. 10.7759/cureus.7492 PMC7193226 32368424

[22] Bright lights in a dark time: Medical students step up to help out | AAMC. cited 2020 55. Available from: https://www.aamc.org/news-insights/bright-lights-dark-time-medical-students-step-help-out

[23] “Itching to get back in”: Medical students graduate early to join the fight | AAMC. cited 2020 55. Available from: https://www.aamc.org/news-insights/itching-get-back-medical-students-graduate-early-join-fight

[24] Soled D, Goel S, Barry D, et al. Medical student mobilization during a crisis: lessons from A COVID-19 medical student response team. Acad Med. 2020 4 8 Published online. DOI:10.1097/ACM.0000000000003401. PMC7188031 32282373

[25] Baker DM, Bhatia S, Brown S, et al. Medical student involvement in the COVID-19 response. Lancet. 2020;395(10232):1254. 32247322 10.1016/S0140-6736(20)30795-9 PMC7270863

[26] Thomson E, Lovegrove S. ‘Let us help’‐ why senior medical students are the next step in battling the COVID‐19 pandemic. Int J Clin Pract. 2020. Published online April 16. DOI:10.1111/ijcp.13516. 32301206

[27] Guidance on Medical Students’ Participation in Direct Patient Contact Activities: April 14, 2020. AAMC. cited 2020 55. Available from: https://www.aamc.org/system/files/2020-04/meded-April-14-Guidance-on-Medical-Students-Participation-in-Direct-Patient-Contact-Activities.pdf

[28] Harris P, Taylor R, Thielke R, et al. Research electronic data capture (REDCap) – A metadata-driven methodology and workflow process for providing translational research informatics support. J Biomed Inf. 2009;42(2):377–381. 10.1016/j.jbi.2008.08.010 PMC2700030 18929686

[29] Harris P, Taylor R, Minor B, et al. The REDCap consortium: building an international community of software partners. J Biomed Inf. 2019. Published online. DOI:10.1016/j.jbi.2019.103208. PMC7254481 31078660

[30] AAMC Medical School Members. AAMC. cited 2020 55. Available from: https://members.aamc.org/eweb/DynamicPage.aspx?site=AAMC&webcode=AAMCOrgSearchResult&orgtype=Medical School

[31] Census Regions and Divisions of the USA. U.S. Census Bureau. cited 2020 55. Available from: https://www2.census.gov/geo/pdfs/maps-data/maps/reference/us_regdiv.pdf

[32] The COVID Tracking Project. cited 2020 55. Available from: https://covidtracking.com/

[33] Maiorova T, Stevens F, Scherpbier A, et al. The impact of clerkships on students’ specialty preferences: what do undergraduates learn for their profession? Med Educ. 2008;42(6):554–562. 18435712 10.1111/j.1365-2923.2008.03008.x

[34] Jones MD, Yamashita T, Ross RG, et al. Positive predictive value of medical student specialty choices. BMC Med Educ. 2018;18:1. 29523127 10.1186/s12909-018-1138-x PMC5845137

[35] Manuel RS. Person-Oriented Versus Technique-Oriented Specialties: early Preferences and Eventual Choice. Med Educ Online. 2009;14. DOI:10.3885/meo.2009.Res00284 20165518 PMC2779627

[36] Ruiz JG, Mintzer MJ, Leipzig RM. The impact of e-learning in medical education. Acad Med. 2006;81(3):207–212. 16501260 10.1097/00001888-200603000-00002

[37] Huynh R. The role of E-learning in medical education. Acad Med. 2017;92(4):430. 10.1097/ACM.0000000000001596 28350599

[38] Stokes DC. Senior medical students in the COVID-19 response: an opportunity to be proactive. Acad Emerg Med. 2020;27(4):343–345. 32215977 10.1111/acem.13972 PMC7161842

[39] Fathy R Coronavirus is keeping Philly medical students out of hospitals, but we are still contributing l Opinion. The Inquirer. cited 2020 58. Available from: https://www.inquirer.com/health/coronavirus/coronavirus-covid19-penn-temple-jefferson-medical-students-20200329.html

[40] Coronavirus in Pennsylvania. Pennsylvania Department of Health. cited 2020 59. Available from: https://www.health.pa.gov/topics/disease/coronavirus/Pages/Coronavirus.aspx

[41] Workbook: NYS-COVID19-Tracker. New York State Department of Health. cited 2020 59. Available from: https://covid19tracker.health.ny.gov/views/NYS-COVID19-Tracker/NYSDOHCOVID- 19Tracker-Map?%3Aembed=yes&%3Atoolbar=no&%3Atabs=n

